# Convergent evolution of viral nucleases targeting cyclic dinucleotides

**DOI:** 10.1371/journal.ppat.1014605

**Published:** 2026-09-15

**Authors:** Zichen Li, Leiliang Zhang

**Affiliations:** 1 Department of Infectious Diseases, Shandong Provincial Hospital Affiliated to Shandong First Medical University, Jinan, Shandong, China; 2 Department of Pathogen Biology, School of Clinical and Basic Medical Sciences, Shandong First Medical University & Shandong Academy of Medical Sciences, Jinan, Shandong, China; University of Iowa, UNITED STATES OF AMERICA

## Abstract

Poxviruses, African swine fever virus (ASFV), and bacteriophages have independently evolved nucleases that degrade cyclic dinucleotides (CDNs), which are critical second messengers that mediate antiviral responses. This Pearl synthesizes recent advancements in our understanding of viral CDN-degrading nucleases and emphasizes three key themes. First, there is the conservation of CDN signaling mechanisms across different biological domains. Second, we observe the diverse structural adaptations of viral nucleases for CDN degradation. Finally, structural protein folds are conserved, allowing for functional replacements across species. Additionally, we discuss the therapeutic potential of targeting these nucleases with resistant CDN analogues and through structure-guided drug discovery. This convergence in viral immune evasion strategies provides valuable insights into the development of broad-spectrum antiviral therapies and offers new opportunities for enhancing immune responses in therapeutic contexts.

## Author summary

Cells have built-in alarm systems to detect viruses. When a virus invades, infected cells produce small signaling molecules called cyclic dinucleotides, or CDNs. These molecules alert nearby cells and trigger immune defenses. Over millions of years, viruses have found ways to disarm this alarm. Large DNA viruses such as poxviruses and African swine fever virus carry enzymes that destroy these danger signals, allowing them to evade the immune system. Bacteria use a similar alarm system to defend against their own viral invaders, phages. Phages have evolved enzymes that destroy bacterial alarm signals. Viruses that infect animals and viruses that infect bacteria have independently arrived at the same solution to the same problem. In this Pearl, we review these recently discovered viral enzymes and show how they share the same basic protein structure despite separate evolutionary paths. We also discuss how understanding these enzymes could aid the development of new antiviral drugs. One strategy is to design molecules that block the viral enzymes and restore immune defenses. Another is to create CDN-like compounds that resist degradation by these enzymes. The convergence of viral immune evasion strategies across different branches of life reveals fundamental principles of host-pathogen conflict and opens new possibilities for therapeutic intervention.

## Introduction

Poxviruses and African swine fever virus (ASFV) are large DNA viruses that replicate in the cytoplasm, leaving their genomes directly exposed to the host DNA sensor cyclic guanosine monophosphate (GMP)-adenosine monophosphate (AMP) (cGAMP) synthase (cGAS). Upon sensing viral DNA, cGAS synthesizes 2′,3′‑cGAMP, which activates STING to induce type I interferons (IFNs) [1]. To avoid this surveillance, these viruses have acquired nucleases, poxin in poxviruses [2–5] and EP364R/C129R in ASFV [6], that degrade 2′,3′‑cGAMP. Remarkably, a similar arms race occurs in bacteria, where the cyclic oligonucleotide‑based antiphage signalling system (CBASS) employs cyclic oligonucleotides (e.g., 3′,3′-cGAMP, 2′,3′-cGAMP, and 3′,3′,3′-cAAA) to trigger infected cell death. Phages, in turn, express phosphodiesterases such as anti-CBASS protein 1 (Acb1) to hydrolyze these bacterial signals [7].

Here we synthesize recent progress on viral CDN‑degrading enzymes, emphasizing three themes: (i) the conserved nature of CDN signaling across domains of life; (ii) the structural and mechanistic solutions evolved by diverse viruses to counter this signaling; and (iii) consistent with the conclusions of Lesk AM and colleagues, conservation of functional protein folds, rather than primary sequence, facilitates cross‑species replacement and presents promising opportunities for drug design [8]. Additionally, we highlight the therapeutic implications of viral CDN-degrading enzymes.

## Cyclic dinucleotide second messengers trigger antiviral responses

**Fig 1:**
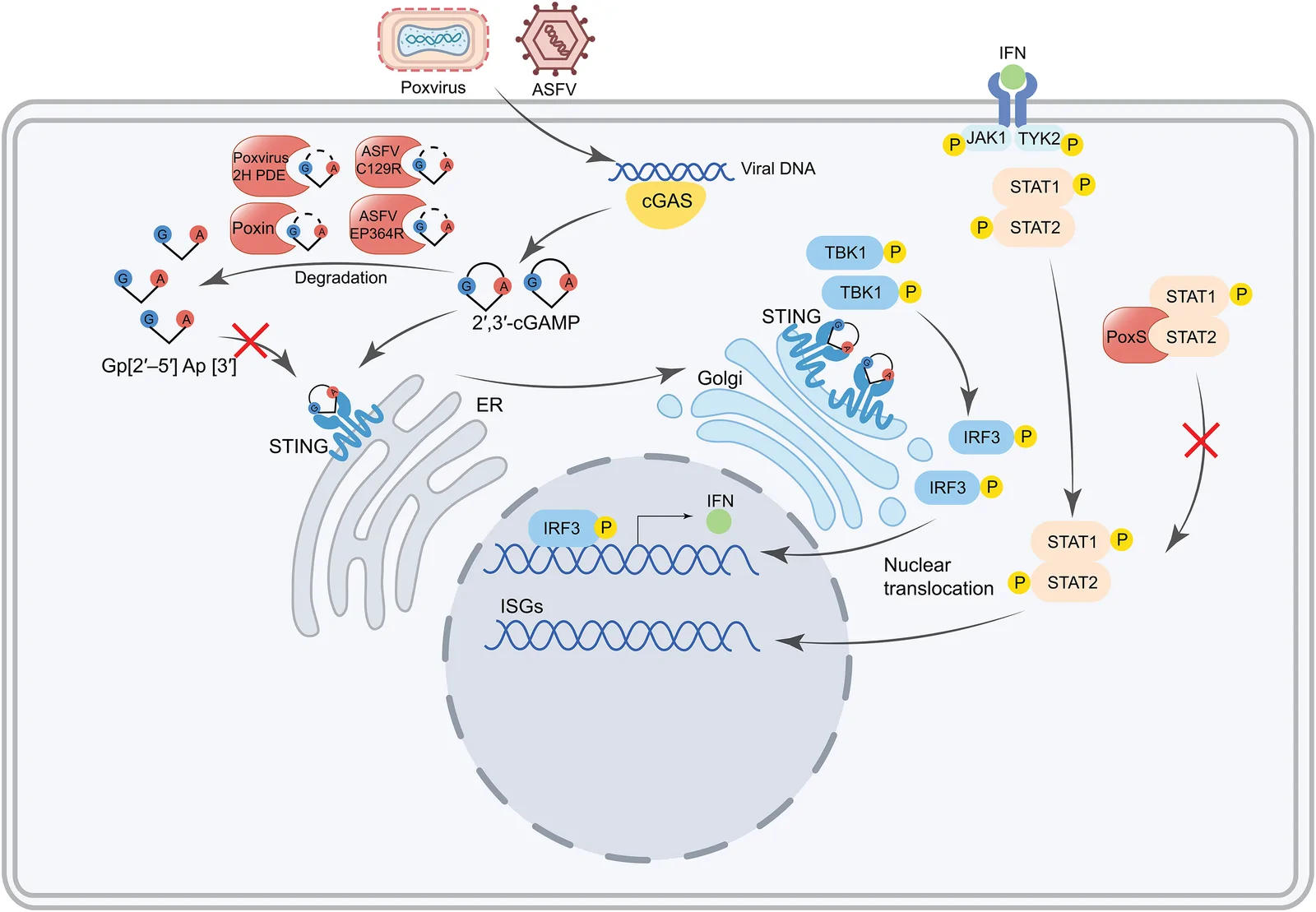
Mechanism of PDEs utilized by viruses to antagonize the cGAS-STING pathway. Upon detecting viral DNA, cGAS synthesizes the second messenger 2′,3′-cGAMP, which binds to STING at the ER membrane. Subsequently, STING migrates to the Golgi, where it recruits and activates TBK1, leading to the phosphorylation and dimerization of IRF3. Phosphorylated IRF3 then translocates to the nucleus to induce the production of IFN. This IFN subsequently activates the JAK-STAT pathway, driving the expression of ISGs. Poxviruses encode poxin or 2H PDE, which hydrolyzes 2′,3′-cGAMP into the linear product Gp[2'-5']Ap[3'], thereby blocking STING activation. In the case of the mpox virus, PoxS specifically binds to STAT2, retaining it in the cytoplasm, which inhibits STAT2 phosphorylation and nuclear translocation, consequently suppressing ISG expression. Additionally, EP364R and C129R from ASFV hydrolyze 2′,3′-cGAMP, thereby inhibiting the cGAS–STING pathway.

In metazoans, cytosolic DNA triggers cGAS to produce 2′,3′‑cGAMP, a unique mixed‑linkage CDN. Notably, some cGAS-like receptors (cGLRs) in different species recognize RNA and synthesize distinct CDNs, for example, 3′,2′-cGAMP in Drosophila [9]. 2′,3′‑cGAMP binds STING, inducing its translocation to the Golgi, recruitment of TBK1, and phosphorylation of IRF3, leading to type I IFN transcription (Fig 1) [1]. Importantly, 2′,3′‑cGAMP can also travel between cells via gap junctions or be packaged into viral particles, acting as a spreading alarm signal.

**Fig 2:**
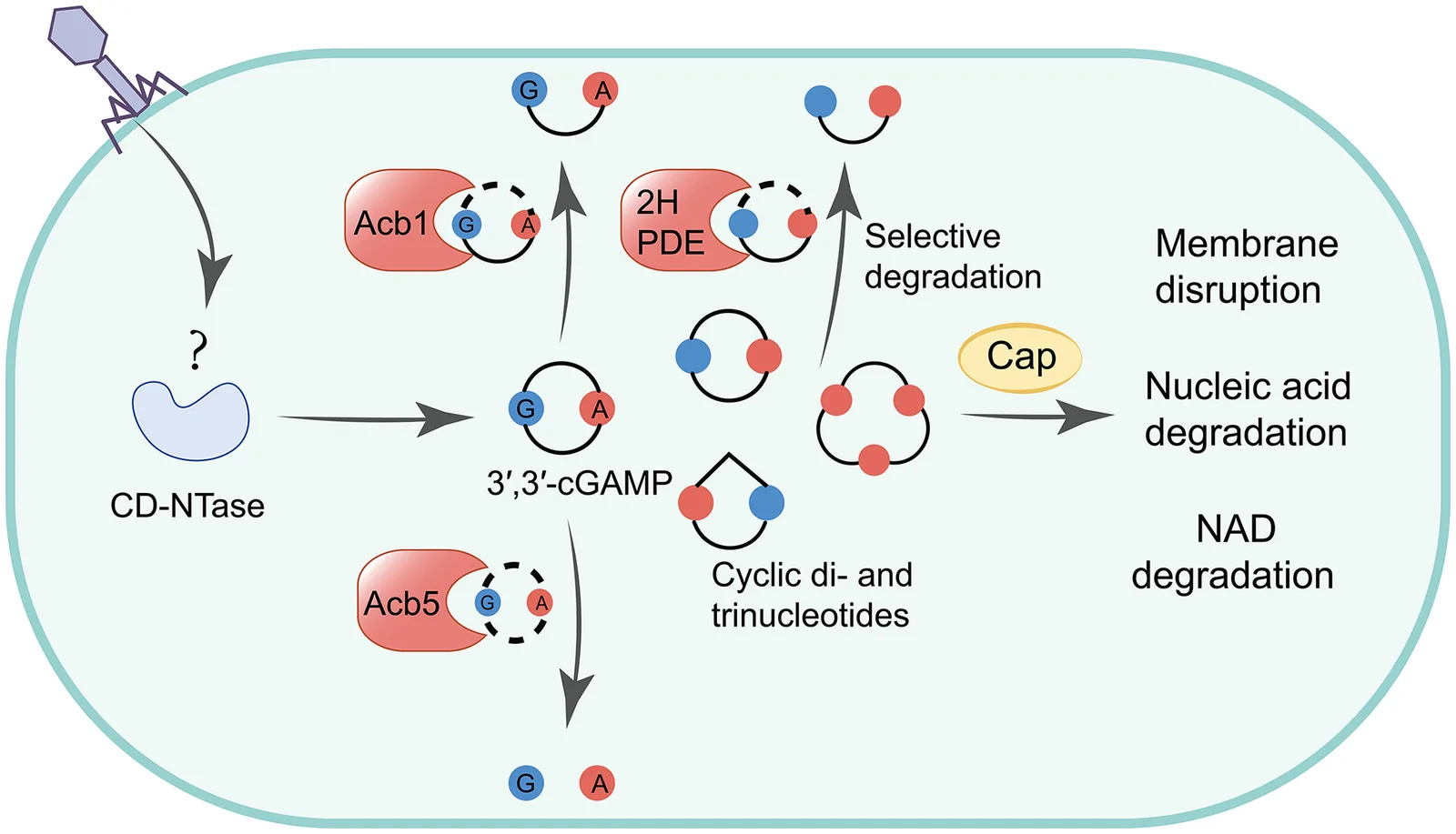
PDEs employed by phages to antagonize the CBASS immune response. In bacteria, CD-NTase detects phage invasion and synthesizes cyclic di- and trinucleotides, activating Cap effectors, which lead to bacterial cell death through membrane disruption, nucleic acid degradation, and NAD degradation. The Acb1 found in phages degrades cyclic di- and trinucleotides containing adenosine into linear products. Acb5 hydrolyzes both 3′-5′ phosphodiester bonds of 3′,3′-cGAMP, producing two single-nucleotide chains: 2′,3′-cAMP and 2′,3′-cGMP. Additionally, other 2H PDEs in phages selectively degrade various cyclic oligonucleotide signals, allowing them to evade bacterial immune responses.

As the origin of cGAS-STING pathway in animal cells, bacteria utilize the CBASS to resist phage infection. cGAS/DncV-like nucleotidyltransferases (CD-NTase) senses phages invasion and catalyzes multiple cyclic oligonucleotide second messengers synthesis, such as 3′,3′-cGAMP and 3′,3′,3′-cAAA, to activate CD-NTase-associated protein (Cap) effectors that execute abortive infection, preventing phage spread (Fig 2) [10]. In some bacterial CBASS pathways, a STING homolog binds cyclic oligonucleotides and drives oligomerization of a TIR effector domain, resulting in rapid NAD^+^ hydrolysis [11]. The structural and enzymatic principles of CD-NTases closely resemble those of animal cGAS, suggesting a shared ancient origin.

Thus, both animals and bacteria rely on CDN second messengers to mount antiviral responses. In response, viruses have evolved dedicated nucleases that destroy these messengers, a classic example of convergent immune evasion.

## Viral CDN‑degrading nucleases suppress host antiviral immunity

Poxin, encoded by the vaccinia virus (VACV) B2R gene, is the first identified viral 2′,3′‑cGAMP nuclease (Fig 1) [2]. It forms a V‑shaped homodimer that binds 2′,3′‑cGAMP in a deep pocket. The catalytic triad His17‑Tyr138‑Lys142 hydrolyzes the 3′‑5′ phosphodiester bond in a metal‑independent manner, yielding linear Guanylyl-(2′ → 5′)-3′-adenylic acid (Gp[2'-5']Ap[3']). Poxin is expressed early during infection, even before genome uncoating, and collaborates with uncoating to reduce cGAS detection [12]. In orthopoxviruses that circulate in rodents (e.g., mpox virus, MPXV; ectromelia virus, ECTV), poxin is fused at its C‑terminus to a viral schlafen (vSlfn) domain, forming PoxS [3,5,13]. This fusion adds an immunosuppressive function: MPXV PoxS cannot fully deplete 2′,3′‑cGAMP below the STING activation threshold, but it instead sequesters STAT2 in the cytoplasm, blocking IFN‑stimulated gene expression [13]. Thus, poxviruses have evolved dual suppression: upstream CDN degradation and downstream STAT2 inhibition. Notably, poxin is inactivated in variola virus and modified VACV Ankara (MVA) [14]. MVA harbors deletions in dozens of immunomodulatory genes, and poxin inactivation only partly accounts for its attenuated phenotype. Notably, the highly virulent variola virus naturally lacks poxin, indicating that poxin loss alone is insufficient for attenuation and that other viral factors critically determine virulence.

ASFV, another cytoplasmic DNA virus, encodes two independent 2′,3′‑cGAMP nucleases (Fig 1) [6]. EP364R contains a STING‑homologous cGAMP‑binding motif (critical residues Tyr76, Asn78) and competitively binds 2′,3′‑cGAMP, while also possessing phosphodiesterase activity that cleaves the molecule [6,15]. C129R acts as a *bona fide* nuclease. Both enzymes reduce intracellular and intercellular cGAMP levels, suppress IFN responses, and are inhibited by the broad‑spectrum phosphodiesterases (PDEs) inhibitor IBMX [6]. Current studies have only functionally validated the nuclease activities of EP364R and C129R. Further structural investigations are required to fully elucidate the molecular mechanisms of these ASFV nucleases. Additionally, ASFV B157L, though lacking catalytic activity, competes with STING for cGAMP binding via its zf‑FCS motif [16]. Hence, ASFV deploys multiple layers of CDN antagonism.

In bacteria, phages have evolved anti‑CBASS proteins. The first described, Acb1 from coliphage T4, is a 2H phosphodiesterase that degrades 3′,3′‑cGAMP and related cyclic nucleotides (3′,3′‑cUA, 3′,3′,3′‑cAAA, and 3′,3′,3′‑cAAG) with a preference for adenosine (Fig 2) [7]. Its structure reveals a U‑shaped pocket formed by six β‑strands and a flexible C‑terminal lid. Substrate binding relies on aromatic residues (Tyr12, Trp74, Phe107, and Trp147) for nonspecific base stacking, while a glutamate (Glu141) hydrogen bonds to adenine N6, explaining the adenosine preference. Upon binding, the lid orders itself and the cyclic nucleotide twists, positioning the 2′‑OH for in‑line attack on the 3′‑5′ bond. The conserved HxT/HxT tetrad (His44, Thr46, His113, and Thr115) catalyzes hydrolysis. More recently, a systematic structural search identified 75 novel viral 2H phosphodiesterases in phages and eukaryotic viruses, grouped into seven families with distinct substrate spectra [17]. Some are broad‑spectrum, while others precisely cleave only specific CDNs. For example, 2H PDE‑9 avoids the bacterial decoy signal 2′,3′‑c‑di‑AMP used by the Panoptes system but efficiently hydrolyzes 3′,3′‑cGAMP, enabling dual escape from both CBASS and Panoptes [18]. Remarkably, entirely new anti‑defence families (e.g., Acb5) were uncovered through AI‑guided structure prediction. Acb5, unlike Acb1, cleaves both 3′‑5′ bonds of 3′,3′‑cGAMP, releasing 2′,3′‑cAMP and 2′,3′‑cGMP (Fig 2) [19]. It forms a homotetramer with double‑symmetric active sites, and residues Trp38, Arg10 and His61 are critical for activity.

Perhaps the most surprising finding is that avian poxviruses, which naturally lack poxin, encode a previously unrecognized cGAMP phosphodiesterase (e.g., gp029 from penguin poxvirus) [20]. Its crystal structure shows near‑identity with the 2H catalytic core of phage Acb1, including the HxT tetrad [21]. Moreover, this avian enzyme can functionally substitute for Acb1 in T4 phage: a recombinant phage in which acb1 was replaced by the avian poxvirus PDE replicated as efficiently as wild‑type, whereas the acb1‑null mutant was severely impaired. This cross‑species complementation demonstrates that the 2H fold is a highly portable module for CDN degradation, and that sequence divergence (often <20% identity) does not preclude functional conservation. Although their overall folds are nearly identical, structural alignments show that the catalytic cores of avian poxvirus PDE and Acb1 are substantially more similar to 2H PDEs from their respective host domains, suggesting the two viral proteins may not share a common ancestor. Moreover, the structure and position of their lid domains differ markedly: phage Acb1 uses a flexible C‑terminal lid to enclose cyclic dinucleotide substrates, whereas the avian poxvirus PDE has an N‑terminal lid formed by an ~20‑residue α‑helical extension. These observations imply that each protein independently acquired additional lid domains to capture nucleotide‑messenger substrates after capture of a host 2H PDE.

## Targeting CDN‑degrading nucleases via resistant analogues and structure-guided discovery

The essential role of CDN‑degrading nucleases in viral immune evasion makes them attractive drug targets. Several 2′,3′‑cGAMP derivatives have been designed to resist poxin hydrolysis while retaining STING agonism. For example: Dideoxy‑2′,3′‑cGAMP lacks the 2′‑ and 3′‑OH groups, removing the nucleophilic attack site; despite lower STING affinity, it shows improved cellular activity due to enhanced metabolic stability [22,23]. Fluorinated carbocyclic cGAMP disrupts hydrogen bonding with poxin His17 and activates all five human STING haplotypes [22]. 5′‑S‑phosphorothioate‑modified cGAMP resists both poxin and the host ectonucleotide pyrophosphatase ENPP1, while maintaining STING activation comparable to ADU‑S100 [23]. These analogues are promising for both antiviral and antitumor immunotherapy.

The crystal structure of MPXV poxin, which is 91% identical to the poxin of the VACV, has facilitated high-throughput virtual screening [24]. Pan and colleagues screened 7,155 small molecules and identified NAD⁺ and theaflavin‑3′‑gallate as competitive binders at the dimer interface of MPXV PoxS [4]. These compounds partially restore cGAS‑STING signaling during MPXV infection, validating poxin as a druggable target. Similar screens could be extended to ASFV EP364R and phage 2H PDEs.

AlphaFold3 and related tools now allow large‑scale structural alignment and binding site prediction, overcoming the limitations of sequence‑based homology searches. The recent discovery of Acb5 and the functional annotation of uncharacterized phage proteins demonstrate that structure‑guided mining of viral genomes will yield many more immune evasion modules [19].

## Conclusion and prospect

From poxviruses to ASFV to phages, viruses have convergently evolved nucleases that target the same class of second messengers, cyclic dinucleotides, albeit synthesized by different host pathways. Nucleases from phages and avian poxviruses exhibit remarkably similar structures: a 2H phosphodiesterase core fold, a conserved catalytic tetrad, and a substrate‑induced lid closure mechanism. This convergence is so strong that an enzyme from an avian poxvirus can fully replace a phage enzyme in a different kingdom of life. Given the greater similarity of each catalytic core to host 2H PDEs and the pronounced differences in lid‑domain structure and location, we propose that these proteins arose via convergent evolution. However, because no intermediate sequences or structures have been identified to date, distant homology cannot be entirely excluded. These findings have profound implications. First, they illustrate that during host‑pathogen arms races, protein fold conservation outlasts sequence divergence; thus, structural biology is indispensable for discovering remote homologes. Second, they suggest that broad‑spectrum antivirals targeting the 2H fold could be effective against both animal and bacterial viruses. For phage applications, Acb1 inhibitors could restore bacterial CBASS antiviral defenses, helping prevent phage contamination in biomanufacturing and aiding control of clinical multidrug‑resistant pathogens. They would also serve as valuable chemical tools for dissecting phage–host immune interactions. Third, the development of poxin‑resistant STING agonists and small‑molecule inhibitors offers a dual therapeutic avenue: enhancing immunity in tumors or restoring immunity in viral infections. Future work should focus on: (i) solving structures of additional viral 2H PDEs to map the determinants of substrate specificity; (ii) developing cell‑permeable inhibitors that target the conserved catalytic pocket; and (iii) exploring whether similar nucleases exist in other cytoplasmic DNA viruses (e.g., marseilleviruses, mimiviruses). The convergence we have described is likely only the tip of the iceberg.

### Ethics statement

This work does not generate original data on human or animal.
